# Clinical and Demographic Characteristics of Patients with Pulmonary Large-Cell Neuroendocrine Carcinoma: A Single-Center Experience

**DOI:** 10.3390/medicina62101980

**Published:** 2026-10-13

**Authors:** Yeşim Özufacık Bölükbaşı, Aydın Aytekin

**Affiliations:** 1 Department of Internal Medicine, Faculty of Medicine, Gaziantep University, 27310 Gaziantep, Türkiye; 2 Division of Medical Oncology, Department of Internal Medicine, Faculty of Medicine, Gaziantep University, 27310 Gaziantep, Türkiye

**Keywords:** pulmonary large-cell neuroendocrine carcinoma, small-cell lung cancer, etoposide, platinum

## Abstract

*Background and Objectives*: Pulmonary large-cell neuroendocrine carcinoma (pLCNEC) is a rare malignancy, accounting for approximately 2–3.5% of surgically resected primary lung cancers. Given its rarity, pLCNEC has been less extensively studied than other, more common lung cancer subtypes. In this study, we evaluated and compared the diagnostic and therapeutic approaches to pLCNEC. *Materials and Methods*: Patients with pLCNEC were evaluated with respect to age, sex, smoking history, primary tumor site, surgical history, tumor stage, histological subtype, metastatic sites at initial diagnosis and during follow-up, first- and second-line chemotherapy regimens, and disease survival parameters. *Results*: A total of 55 patients diagnosed between 2012 and 2024 were retrospectively analyzed. The median age at diagnosis was 63 years, the mean smoking exposure was 45.8 pack-years, and the median overall survival (mOS) for the entire cohort was 9 months (95% CI, 7–11). At presentation, 12.7% of the patients had stage I–II, 29.1% had stage III, and 58.2% had stage IV disease. Primary tumors were located peripherally in 85.5% of cases and centrally in 14.5%. A platinum plus etoposide regimen—standard for small-cell lung cancer (SCLC)—was the most commonly administered first-line chemotherapy (*n* = 40). Among all patients, 19 had undergone upfront surgical resection, whereas 36 had not. In univariate analysis, patients with a history of surgery (*n* = 19) had a significantly longer mOS than those without surgery (*n* = 36): 16 months (95% CI, 8–NR) versus 7 months (95% CI, 3–9), respectively (*p* = 0.003). On multivariate analysis, stage IV disease, ECOG PS ≥ 2, and advanced age were independently associated with poorer survival. In multivariable analyses, surgery showed a borderline association with survival that did not reach statistical significance (*p* = 0.052). Furthermore, median survival was significantly longer in patients with T1 stage disease compared with those with T3 and T4 stage, and in those with T3 compared with T4 stage (*p* = 0.045). *Conclusions*: Overall, early-stage diagnosis and surgical intervention may be associated with favorable clinical outcomes and a survival benefit.

## 1. Introduction

Although pulmonary neuroendocrine tumors have been classified heterogeneously into various groups and categories over the years, they are currently divided into four major groups: atypical carcinoid, typical carcinoid, small-cell lung cancer (SCLC), and large-cell neuroendocrine carcinoma (LCNEC) [1]. Low-grade typical and atypical carcinoid tumors are generally associated with a favorable prognosis and low metastatic potential [2,3]. In contrast, SCLC exhibits a highly aggressive clinical course and is generally associated with a poor prognosis [4]. Pulmonary LCNEC (pLCNEC) is a rare entity, representing approximately 1% of all resected lung cancer cases [5]. The overall incidence of resectable pLCNEC is estimated to be less than 3%, with long-term survival rates (3-year and 5-year) ranging from 21% to 57% [6,7]. Similar to SCLC, pLCNEC is considered a high-grade neuroendocrine disease that is associated with a poor prognosis and a higher incidence among smokers [8]. Morphologically, LCNEC is characterized by neuroendocrine architecture (e.g., rosette-like structures, nesting patterns), moderate to abundant cytoplasm, frequently prominent nucleoli, a high mitotic rate (>10 per mm^2^)/Ki-67 labeling index, and the presence of necrosis [9]. Diagnosis requires immunohistochemical confirmation of at least one neuroendocrine marker (e.g., synaptophysin, chromogranin, and CD56) in ≥10% of tumor cells [10]. Clinically, LCNEC is typically associated with a high rate of metastasis and poor patient survival, although widely variable survival outcomes have been reported in the literature [11]. Additionally, highly variable outcomes have been reported regarding the chemosensitivity of pLCNEC to platinum/etoposide-based regimens used for SCLC [12,13,14], resulting in a lack of consensus on whether pLCNEC should be managed as non-small-cell lung cancer (NSCLC) rather than SCLC [12,13,14]. With current diagnostic capabilities allowing LCNEC to be distinguished from other histotypes, it has become evident that these tumors generally exhibit a more aggressive behavior than other NSCLCs [2,15]. Another histological variant in the differential diagnosis is large-cell carcinoma. Large-cell carcinoma is an undifferentiated NSCLC lacking light-microscopic evidence of squamous or glandular differentiation; however, squamous or glandular features may be detectable upon ultrastructural examination [16]. Due to its rarity and the persistent challenges in differential diagnosis, both the prognosis and optimal management of this group of malignancies remain controversial [17,18]. As a rare malignancy, it has been less extensively studied in the literature compared with other common lung cancer subtypes. The majority of available studies are retrospective and limited by small sample sizes. In the present study, we aimed to evaluate the descriptive clinical and demographic characteristics of patients diagnosed with pLCNEC.

## 2. Materials and Methods

This retrospective study was conducted at Gaziantep University Şahinbey Practice and Research Hospital with the approval of the Gaziantep University Clinical Research Ethics Committee (dated 13 March 2024, decision no: 2024/52). The study was conducted in accordance with the principles of the Declaration of Helsinki and the regulations on patient rights. A total of 55 patients diagnosed with pLCNEC were included in the study.

Inclusion Criteria: the inclusion criteria were age ≥18 years, a confirmed pathological diagnosis, and a diagnosis of pLCNEC. Exclusion Criteria; the exclusion criteria were age <18 years, the absence of a pathological diagnosis, and histological subtypes for which a definitive distinction could not be established on pathological examination.

Study Protocol: Patient demographics and clinical parameters were evaluated, including sex, age, smoking status, primary tumor location, initial stage, post-diagnostic surgical status, history of adjuvant chemotherapy and/or radiotherapy, Ki-67 proliferation index and the presence of gene mutations on pathological examination, Eastern Cooperative Oncology Group (ECOG) performance status score, organ or tissue metastases, therapeutic regimens, post-treatment response assessments according to RECIST criteria, imaging modalities utilized during follow-up, number of treatment lines received, post-treatment progression status, and overall survival outcomes. Mixed histology refers to a pathological condition where LCNEC is the dominant pathology and constitutes at least 50% of the surgical specimen.

Statistical Analysis: In this study, variables comprising demographic data and clinical parameters from 55 participants were analyzed using IBM SPSS Statistics software, version 23 (IBM Corp., Armonk, NY, USA). The normality of continuous variables was assessed using the Shapiro–Wilk test. As the data did not show a normal distribution, continuous variables were presented as median (minimum–maximum) values, while categorical variables were presented as frequencies and percentages. Correlations between two continuous variables were evaluated using the Spearman correlation coefficient. The Mann–Whitney U test was used to compare continuous (time-independent) variables between two independent groups, whereas the Kruskal–Wallis test (accompanied by pairwise post hoc comparisons where appropriate) was used for comparisons among more than two groups. Overall survival (OS) was defined as the time from the date of pathological diagnosis to death from any cause or the date of last follow-up and was estimated using the Kaplan–Meier method; median survival times were reported with 95% confidence intervals. Dates of death for patients lost to follow-up were obtained from the “death notification system.” Differences in OS between subgroups defined by categorical variables were compared using the log-rank test. Variables found to be associated with OS at the *p* < 0.05 level in univariate log-rank analysis were included in a multivariate Cox proportional hazards regression model to identify independent prognostic factors; results were reported as hazard ratios (HR) with 95% confidence intervals. For all statistical analyses, a *p*-value of <0.05 was considered statistically significant.

## 3. Results

The median age of the 55 patients was 63 years (range, 41–89). Four patients (7.3%) were female, and 51 patients (92.7%) were male. The median ECOG performance score was 1. Five patients (9.1%) were never-smokers, 48 patients (87.3%) were former smokers, and two patients (3.6%) were current smokers. The mean smoking exposure was 45.82 pack-years (range, 0–120). According to the initial disease stage, 12.7% (*n* = 7) of patients had early-stage disease (stages I–II), 29.1% (*n* = 16) had locally advanced disease (stage III), and 58.2% (*n* = 32) had advanced-stage disease (stage IV) (Table 1).

When TNM staging was evaluated, 2 patients (3.6%) had T1, 15 (27.3%) had T2, 19 (34.5%) had T3, and 19 (34.5%) had T4 tumors. Regarding nodal status, 13 patients (23.6%) were N0, 16 (29.1%) were N1, 16 (29.1%) were N2, and 10 (18.2%) were N3. Thyroid transcription factor-1 (TTF-1) expression was negative in 31 patients (56.4%), positive in 23 patients (41.8%), and unknown in 1 patient (1.8%). Furthermore, synaptophysin was negative in 10 patients (18.2%) and positive in 45 patients (81.8%), whereas chromogranin was negative in 24 patients (43.6%), positive in 30 patients (54.5%), and unknown in 1 patient (1.8%). The Ki-67 index was assessed in 31 of the 55 patients. The mean Ki-67 proliferation index was 72.74% (range, 30–90%). Standard molecular testing for NSCLC (EGFR, ALK, ROS-1) was performed in 9 patients (16.36%), and an ALK gene rearrangement was identified in 1 patient. At initial diagnosis, 36 patients (65.5%) had no surgical history, while 19 patients (34.5%) had undergone surgery. Of the 23 patients with early-stage and locally advanced disease, 19 (82.6%) received postoperative chemotherapy. Five patients (9.1%) presented with mixed histology, whereas the remaining patients had pure pLCNEC histology (Table 1).

Regarding the primary tumor location, 19 patients (34.5%) had tumors in the right upper lobe, 5 (9.1%) in the right lower lobe, 15 (27.3%) in the left upper lobe, 11 (20.0%) in the left lower lobe, and 5 (9.1%) in other anatomical locations. The primary tumor was located peripherally in 47 patients (85.5%) and centrally in 8 patients (14.5%). Endobronchial lesions were absent in 52 patients (94.5%) and present in 3 patients (5.5%) (Table 1).

Regarding metastatic distribution at first-line treatment, central nervous system (CNS) metastases were detected in 9 patients (16.4%), liver metastases in 12 (21.8%), bone metastases in 19 (34.5%), adrenal metastases in 9 (16.4%), and distant lymph node metastases in 35 (63.6%). At the time of the study analysis, 12 patients (21.8%) were alive, while 43 patients (78.2%) had died (Table 2).

Regarding first-line treatment regimens, an SCLC-based regimen was selected for 40 patients (%72.7%), whereas an NSCLC-based regimen was selected for 8 patients 14.5%). Seven patients (12.7%) had an unknown treatment status or had not received treatment. Among the SCLC-based regimens, etoposide plus cisplatin was administered to all 40 patients. The NSCLC-based regimens included vinorelbine plus cisplatin in 2 patients, docetaxel in 2 patients, pemetrexed in 1 patient, carboplatin plus paclitaxel in 2 patients, and gemcitabine in 1 patient. There were no patients receiving immunotherapy in our study cohort.

Among the participants, the best overall response according to RECIST criteria during first-line treatment was complete response (CR) in 5 patients (9.1%), partial response (PR) in 8 patients (14.5%), stable disease (SD) in 1 patient (1.8%), and progressive disease (PD) in 20 patients (36.4%). The response was not assessed or was unknown in 21 patients (38.2%). Regarding the reasons for discontinuation of first-line treatment, 7 patients (12.7%) did not receive the treatment course, 31 patients (56.3%) [19 (34.5%) discontinued because of disease progression, 12 (21.8%) because of death] had disease progression, and 4 (7.3%) for unknown reasons. Eleven patients (20.0%) completed the treatment course, while 2 patients (3.6%) were still receiving it (Table 2).

A total of 17 patients received second-line treatment. Due to patient mortality or loss to follow-up following first-line therapy, evaluations were conducted on these 17 patients. Regarding second-line treatment regimens, an SCLC-based regimen was preferred in 11 patients (64.7%), whereas an NSCLC-based regimen was selected in 6 patients (35.3%). Among the SCLC-based regimens, etoposide plus cisplatin was administered to 5 patients, irinotecan to 3, topotecan to 2, and cisplatin plus irinotecan to 1. The NSCLC-based regimens included carboplatin plus paclitaxel in 5 patients and docetaxel in 1 patient (Table 2).

Regarding the overall distribution of second-line treatment regimens, 38 patients (69.1%) did not receive second-line therapy, 5 (9.1%) received platinum plus etoposide, 3 (5.5%) irinotecan, 5 (9.1%) carboplatin plus paclitaxel, 2 (3.6%) topotecan, 1 (1.8%) docetaxel, and 1 (1.8%) cisplatin plus irinotecan. In terms of best overall response according to RECIST criteria in the second-line setting, (CR) and (SD) were achieved in 1 patient (1.8%) each, while (PD) was observed in 8 patients (14.5%); response was not assessed or was unknown in 45 patients (81.8%). Finally, 22 patients (40.0%) were lost to follow-up, and 33 patients (60.0%) were actively followed (Table 2).

**Figure 1:**
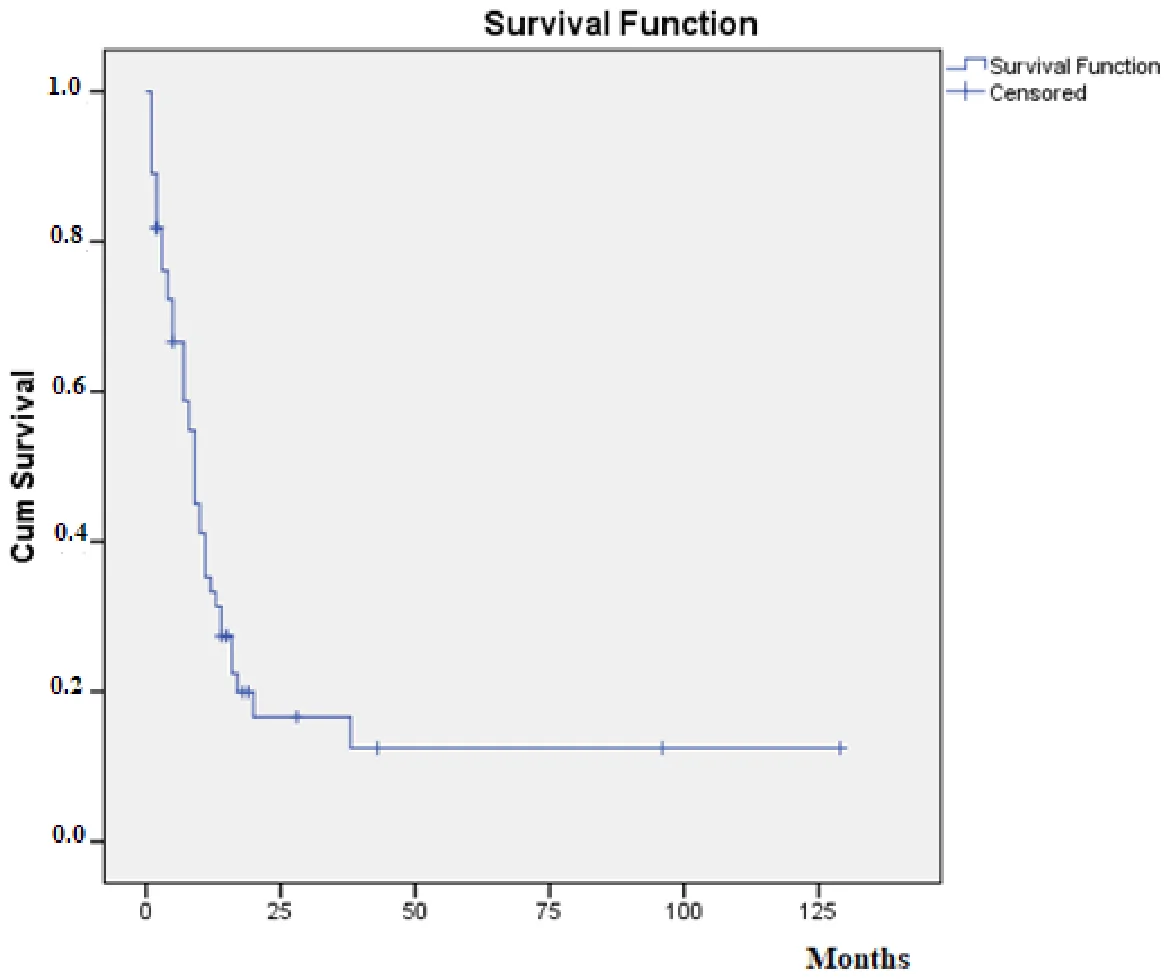
Kaplan–Meier curve of overall survival for the entire cohort (*n* = 55).

**Figure 2:**
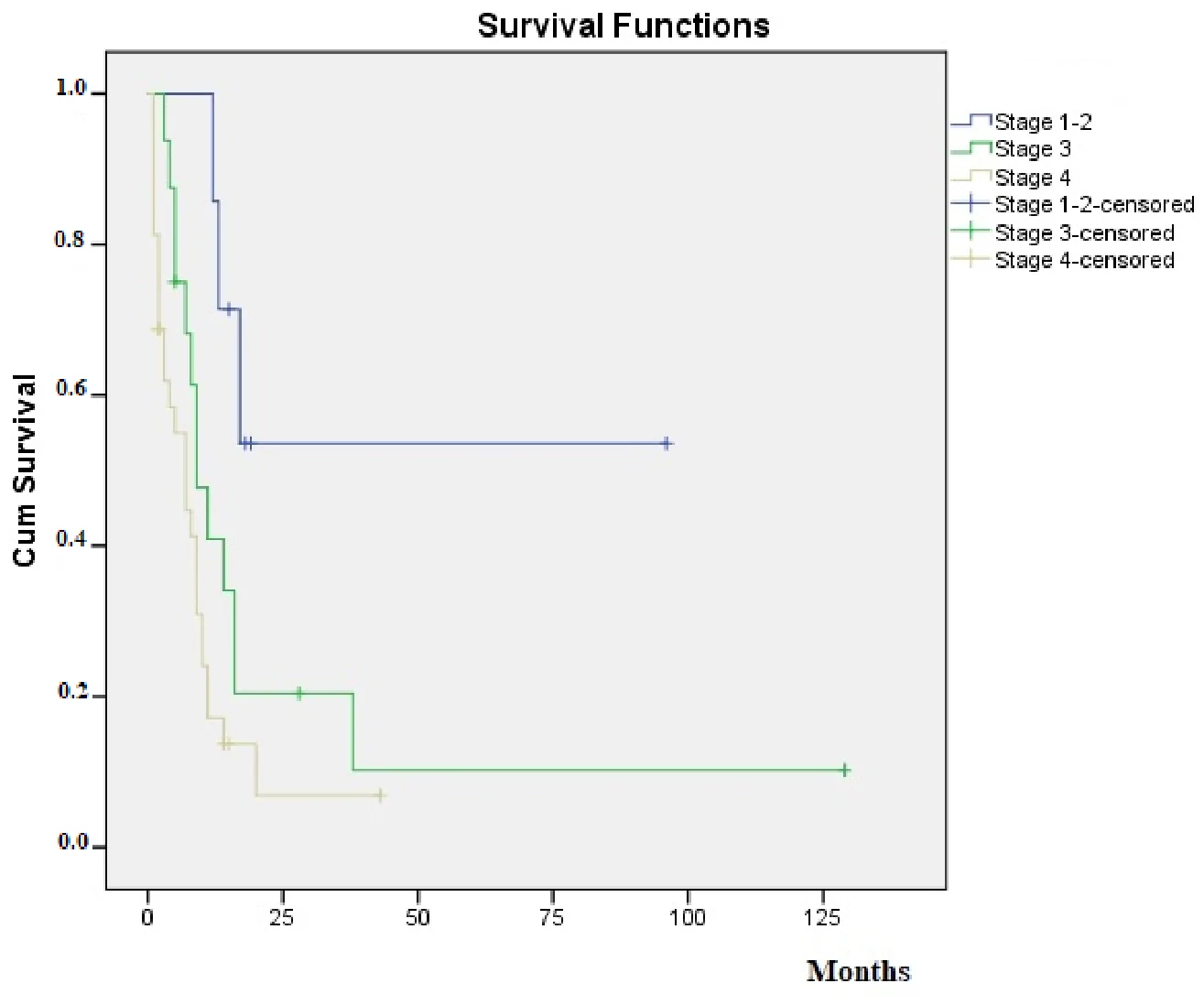
Kaplan–Meier overall survival curves by disease stage.

Median overall survival (mOS) for the entire cohort was 9 months (95% CI, 7–11) (Figure 1). The mOS was not reached (NR) among the seven patients with stage I–II disease, owing to the small number of cases. The mOS was 9 months (95% CI, 5–16) in patients with stage III disease (*n* = 16) and 7 months (95% CI, 2–9) in those with stage IV disease (*n* = 32) (Figure 2) (Table 3). Overall survival (OS) differed significantly across stages (log-rank, *p* = 0.005). The mOS was not reached among the two patients with T1 disease. In patients with T2 (*n* = 15), T3 (*n* = 19), and T4 disease (*n* = 19), the mOS was 11 months (95% CI, 2–NR), 9 months (95% CI, 5–14), and 7 months (95% CI, 3–9), respectively. Survival differed significantly among the T-stage groups (log-rank *p* = 0.045). By N stage, the mOS was 13 months (95% CI, 4–NR) for N0 (*n* = 13), 10 months (95% CI, 4–12) for N1 (*n* = 16), 9 months (95% CI, 5–14) for N2 (*n* = 16), and 2 months (95% CI, 1–9) for N3 disease (*n* = 10). The difference among N-stage groups was not statistically significant (*p* = 0.172). The mOS was 14 months (95% CI: 9–38) in M0 patients compared to 7 months (95% CI: 2–9) in M1 patients (*p* = 0.004). Among patients with negative (*n* = 31) and positive TTF-1 expression (*n* = 23), the mOS was 8 months (95% CI, 4–10) and 11 months (95% CI, 5–38), respectively. The difference did not reach statistical significance (*p* = 0.092). Among patients with negative (*n* = 10) and positive synaptophysin expression (*n* = 45), the mOS was 7 months (95% CI, 4–14) and 9 months (95% CI, 5–12), respectively, with no statistically significant difference between the groups (*p* = 0.639). Similarly, the mOS was 8 months (95% CI, 4–16) in patients with negative chromogranin expression (*n* = 24) and 9 months (95% CI, 5–11) in those with positive expression (*n* = 30); the difference was not statistically significant (*p* = 0.977). No statistically significant difference in survival was observed among the groups defined by primary tumor location (*p* = 0.846) (Table 4).

Patients with a history of surgery (*n* = 19) had a significantly longer mOS than those without surgery (*n* = 36): 16 months (95% CI, 8–NR) versus 7 months (95% CI, 3–9), respectively (*p* = 0.003). Age (years) was significantly associated with survival (HR, 1.056; 95% CI, 1.021–1.093; *p* = 0.0015), and patients were accordingly stratified using a cutoff of 65 years. The mOS was 14 months (95% CI, 10–20) in patients younger than 65 years (*n* = 30) compared with 5 months (95% CI, 3–7) in those aged 65 years or older (*n* = 25), a statistically significant difference (*p* < 0.001). When survival was analyzed according to the RECIST response after the first line of chemotherapy, the mOS was not reached (NR; 95% CI, 13–NR) in patients with complete response (*n* = 5), 7 months (95% CI, 3–NR) in those with partial response (*n* = 8), NR in the single patient with stable disease (*n* = 1), 10 months (95% CI, 7–14) in those with disease progression (*n* = 20), and 4 months (95% CI, 1–8) in patients who were not evaluable (*n* = 21); the difference among these groups was statistically significant (*p* = 0.0005). This difference may be attributable to the fact that patients lost to follow-up had generally died, whereas patients with disease progression continued on to second-line chemotherapy, introducing a form of immortal time (guarantee-time) bias. No statistically significant association with survival was found for smoking amount (HR, 1.005; 95% CI, 0.995–1.015; *p* = 0.341) or Ki-67 index (HR, 1.010; 95% CI, 0.984–1.037; *p* = 0.471).

On multivariate analysis, stage IV disease, ECOG PS ≥ 2, and advanced age were independently associated with poorer survival. When factors such as stage, ECOG performance status, and age were evaluated, stage III disease showed a near-significant association compared with stage I–II disease (HR, 3.18; 95% CI, 0.89–11.35; *p* = 0.075), whereas stage IV disease was significantly associated with survival compared with stage I–II disease (HR, 4.06; 95% CI, 1.18–13.97; *p* = 0.026). When the effect of surgery was evaluated alongside T stage, N stage, and ECOG performance status, Cox regression analysis revealed a near-significant independent association (*p* = 0.052). In contrast, age (≥65 years) and ECOG PS (≥2) emerged as the strongest and most consistent prognostic factors, independent of stage and surgical status. In conclusion, the survival difference between patients who did and did not undergo surgery approached, but did not exceed, the conventional threshold for statistical significance (*p* = 0.052) when evaluated independently of stage (with T stage, N stage, age, and performance status controlled for).

## 4. Discussion

Although research and therapeutic options regarding pLCNEC remain limited, recent studies focusing on the clinical characteristics and prognosis of pLCNEC have increasingly been published. Lowczak et al. demonstrated that, similar to SCLC, pLCNEC is predominantly associated with the male sex [19]. Similarly, another study conducted by Chen et al. involving patients with metastatic pLCNEC reported that pLCNEC was more common among males [20]. In our study, 51 of the 55 patients (92.7%) were male. Consistent with the literature, these descriptive findings from our cohort demonstrate a strong association between pLCNEC and the male sex.

Fisseler et al. reported that pLCNEC tumors were located peripherally in 84% of cases and centrally in 16%. They also noted that peripherally located tumors could not be reached by bronchoscopy, whereas centrally located tumors developed with endobronchial tumor growth [21]. In our study, a similar distribution was observed: 85.5% of primary tumors were peripherally located and 14.5% were centrally located. These findings highlight that transbronchial biopsy techniques are at least as important as endobronchial methods in the diagnostic evaluation of pLCNEC.

Previous studies have demonstrated that the biological behavior of pLCNEC tumors closely resembles that of small-cell lung carcinomas [18,22,23]. Because the clinicopathological and biological characteristics of pLCNEC closely resemble those of small-cell lung carcinoma, Iyoda et al. initiated a prospective study in 2000 evaluating these patients with cisplatin plus etoposide, a chemotherapy regimen standardly used for small-cell lung carcinoma [24]. The results demonstrated a favorable prognosis in patients receiving cisplatin plus etoposide-based chemotherapy. Furthermore, in a retrospective study, Rossi et al. reported that platinum plus etoposide-based adjuvant chemotherapy was effective in patients with pLCNEC [13]. In our retrospective study, analysis of first-line treatment regimens revealed that an SCLC-based regimen (platinum plus etoposide) was selected in 40 patients (72.7% whereas an NSCLC-based regimen was preferred in 8 patients (14.5% In clinical practice, therapeutic strategies are frequently formulated based on the Ki-67 proliferation index; particularly when the Ki-67 index exceeds 50–60%, tumors are managed as SCLC, and platinum plus etoposide regimens are preferentially selected. However, sufficient scientific evidence supporting this approach remains lacking. In our cohort, no statistically significant relationship was identified between the Ki-67 index and overall survival.

Furthermore, adequate scientific evidence is currently lacking regarding whether immunotherapy agents that have become standard of care in SCLC (such as atezolizumab and durvalumab) should be combined with platinum plus etoposide regimens in the treatment of pLCNEC. In our study, the SCLC-based protocol consisted of etoposide plus cisplatin (*n* = 40), while the NSCLC-based regimens included vinorelbine plus cisplatin (*n* = 2), docetaxel (*n* = 2), pemetrexed (*n* = 1), carboplatin plus paclitaxel (*n* = 2), and gemcitabine (*n* = 1). No patients in our cohort received immunotherapy. Evaluation of first-line therapeutic responses demonstrated complete response in 5 patients, partial response in 8, stable disease in 1, progressive disease in 20, and not assessed/unknown status in 21. These findings suggest that pLCNEC is a highly aggressive tumor and underline the need for more effective first-line chemotherapy regimens in the future.

In the study conducted by Rossi et al. in 2005 [13], the primary metastatic sites were the brain, bone, and liver. These metastatic patients received a platinum plus etoposide chemotherapy regimen, yielding an overall response rate of 29%. Complete response was achieved in 2 patients, and partial response was observed in 4 patients. In contrast, no complete or partial responses were observed among patients treated with other chemotherapy regimens. In our cohort, prior to first-line therapy, bone metastases were present in 34.5% (*n* = 19), liver metastases in 21.8% (*n* = 12), and (CNS) metastases in 16.4% (*n* = 9) of the patients. Distant lymph node metastases were identified in 35 patients (63.6%) before first-line treatment. Patients presenting with stage IV disease accounted for 58.2% (*n* = 32) of the entire study cohort at the time of diagnosis. These findings indicate that a high proportion of pLCNEC cases present with distant metastatic disease at initial diagnosis. Consistent with the literature, cisplatin plus etoposide—an SCLC-based regimen—was predominantly administered to these patients in our clinical practice.

In the study conducted by Derks et al. [25] involving 952 patients diagnosed with pLCNEC, the (mOS) times for stages I–II combined was 32.4 months (22.0–42.9), for stage III were 12.6 months (10.3–15.0), and for stage IV disease were 4.0 (3.5–4.6) months. In our study, mOS could not be reached in patients with stage 1–2 disease due to the small number of patients. The mOS 9 months (95% CI: 5–16) for stage III patients, and 7 months (95% CI: 2–9) for stage IV patients. When evaluated according to T stage, mean survival was significantly longer in patients with T1 stage disease compared with those with T3 and T4 stage disease, and patients with T3 stage disease survived significantly longer than those with T4 stage disease (*p* = 0.045). These findings suggest that advancing T stage is associated with a progressively worse prognosis.

Eichhorn et al. [5] demonstrated that advanced nodal involvement (N1, N2), higher disease stage, and the presence of metastases were associated with tumor-related death and earlier disease recurrence. Additional studies have also reported that early-stage TNM classification and the absence of distant metastases correlate favorably with overall survival [26,27]. Similarly, when comparing M0 patients (*n* = 23) with M1 patients (*n* = 32), we identified a statistically significant survival advantage in M0 patients compared with M1 patients (*p* = 0.004). When pLCNEC is diagnosed at an early stage (stages I and II), surgical resection may be recommended to achieve a more favorable prognosis [28]. In a study by Gu et al. [28], where patients were stratified into surgical and non-surgical cohorts, surgical resection was shown to confer a survival benefit in patients with stage I, II, and III pLCNEC. In our study, univariate analysis revealed that patients with a history of surgery (*n* = 19) achieved significantly longer median overall survival (mOS) compared to those without surgery (*n* = 36): 16 months (95% CI: 8–NR) versus 7 months (95% CI: 3–9), respectively (*p* = 0.003). However, in multivariable analysis, surgery demonstrated only a borderline association with survival that did not reach statistical significance (*p* = 0.052). Despite these statistical findings, several retrospective studies have reported that surgical resection alone is insufficient even in early-stage disease and that adjuvant chemotherapy should be administered following surgical intervention [22,29,30]. Despite the poor prognosis associated with this malignancy, surgical resection should be considered whenever feasible due to its possible favorable contribution to overall survival, even if complete cure is rarely achieved.

In the literature, an adequate number of clinical studies evaluating second- and third-line therapies in metastatic pLCNEC have not been reported. Two studies investigated amrubicin monotherapy as a second-line treatment in pLCNEC. The first study enrolled 18 patients (13 males and 5 females) with pLCNEC or “high-grade non-small cell neuroendocrine carcinoma,” all of whom had previously received platinum-based first-line therapy. In that study, single-agent amrubicin yielded an overall response rate (ORR) of 11.1%, a median progression-free survival (mPFS) of 4.0 months, and an mOS of 9.1 months [31]. Another retrospective study similarly evaluated amrubicin in advanced pLCNEC following disease progression on platinum-based chemotherapy. Among these patients, 72% had received one line of chemotherapy, whereas the remainder had received two or more prior lines. A total of 18 patients (17 males and 1 female) were included in the study, yielding an ORR of 28%, a median progression-free survival (mPFS) of 3.1 months, and an mOS of 5.1 months [32].

Levra et al. [33] reported an impressive ORR of 60% in a case series of 10 patients with advanced pLCNEC treated with single-agent nivolumab or pembrolizumab in the second-line setting. In that study, the mPFS was 14 months. In our study, there were no patients receiving immunotherapy in our cohort. The second-line treatment regimens administered included platinum plus etoposide (*n* = 5), irinotecan (*n* = 3), carboplatin plus paclitaxel (*n* = 5), topotecan (*n* = 2), docetaxel (*n* = 1), and cisplatin plus irinotecan (*n* = 1). Regarding treatment responses, complete response was observed in 1 patient, stable disease in 1 patient, and progressive disease in 8 patients, whereas response could not be assessed in 45 patients. An evaluation of the current literature alongside our findings highlights the urgent need for more effective second- and third-line systemic therapy regimens in metastatic pLCNEC. In our cohort, during second-line and subsequent treatment regimens, soft tissue was the most common site of metastasis in 8 patients.

Despite significant advancements in understanding the molecular landscape of lung adenocarcinoma, pLCNEC remains poorly characterized to date due to its low prevalence. Nonetheless, given its aggressive clinical features, several studies have investigated the molecular profile of pLCNEC. Although chromogranin-A represents the most specific molecular marker in pLCNEC with a positivity rate of approximately 70%, its sensitivity remains relatively low. Conversely, synaptophysin is expressed in up to 87% of pLCNEC cases; however, its diagnostic specificity is limited, as it is also detectable in approximately 10% of lung adenocarcinomas and 5% of squamous cell carcinomas [34]. The expression of these markers is largely dependent on the degree of differentiation, and diagnostic confirmation requires positive staining for at least one of the aforementioned antibodies in at least 50% of the malignant cells [35]. Thyroid transcription factor 1 (TTF-1) is expressed in approximately 41–75% of pLCNEC cases [36]. In our study, TTF-1 positivity was identified in 41.8% of patients (*n* = 23), synaptophysin in 81.8% (*n* = 45), and chromogranin in 54.5% (*n* = 30). While our findings regarding TTF-1 and synaptophysin expressions are consistent with the published literature, chromogranin positivity was detected at a lower rate in our cohort. Consequently, the sensitivity of chromogranin in pLCNEC was observed to be lower in our study compared with previously reported data.

In a study comprising 94 patients, immunohistochemical analysis revealed oncogenic driver alterations, including EGFR mutations (8.3%) and ROS-1 and ALK rearrangements (5.8%) [37]. In a study conducted by Zhou et al. [38] involving 126 patients with pLCNEC, a ROS1 gene rearrangement was identified in one patient with a peripherally located tumor. Among the patients included in our study, ALK, EGFR and ROS-1 testing was performed in 9 patients. An ALK gene rearrangement was identified in one patient, whereas EGFR and ROS1 testing did not identify eligibility for targeted therapy. Molecular mutation screening was not performed in 46 patients. The high proportion of patients who did not undergo molecular mutation testing is likely attributable to the fact that pLCNEC is predominantly managed as SCLC in clinical practice, leading to the preferential selection of SCLC-directed therapeutic regimens.

pLCNEC is strongly associated with a history of tobacco smoking [39]. In previously published studies on pLCNEC, almost all patients were active or former smokers [40]. Among the patients included in our study, 5 (9.1%) were never-smokers, 48 (87.3%) were former smokers, and 2 (3.6%) were current smokers. The mean smoking exposure of the 55 patients was 45.82 pack-years. The occurrence of pLCNEC in five never-smokers represents a rare clinical entity, underscoring the clear need for further research to elucidate the underlying pathogenesis of this disease. The Ki-67 proliferation index was evaluated in 31 patients, yielding a mean value of 72.74%. No statistically significant correlation was observed between mOS and smoking exposure or Ki-67 proliferation index (*p* > 0.05).

This study has several limitations. First, the small sample size (*n* = 55), retrospective design, and single-center nature restrict the statistical power and generalizability of the findings. Second, missing data and a high rate of loss to follow-up introduced potential confounding factors and immortal time bias. Third, due to the limited number of early-stage patients, we could not perform a adequately powered head-to-head comparison between surgically resected and non-resected cohorts with comparable stages and baseline characteristics; this likely contributed to the overlap in survival curves between surgical history and overall stage, as well as the lack of statistically definitive differences regarding the survival impact of surgery. Fourth, a substantial attrition rate occurred from second-line therapy onward, largely driven by mortality or loss to follow-up, the latter being compounded by our institution serving as a regional tertiary referral center where patients frequently transitioned to local facilities following initial diagnosis and treatment planning. Finally, the number of patients who underwent genetic profiling was limited, and treatment response evaluation per RECIST criteria could not be determined in a subset of patients who were lost to follow-up.

## 5. Conclusions

pLCNEC is a rare subtype of lung cancer predominantly seen in males, typically characterized by peripheral tumor localization, high Ki-67 proliferation index, frequent positivity for neuroendocrine markers, and a low anticipated burden of oncogenic driver alterations. In clinical practice, SCLC-directed chemotherapy protocols are frequently preferred, whereas second-line and subsequent therapeutic options remain limited and inadequate. Although surgical resection demonstrated a trend toward improved survival in early stages, the disease carries an inherently poor prognosis with short overall survival. Consequently, large-scale, randomized controlled trials are urgently needed to elucidate its pathogenesis, characterize comprehensive molecular and genetic profiles, and establish optimal treatment strategies.

## Figures and Tables

**Table 1:** Clinical and Demographic Findings—1.

Variables	Categories	(*n*)	(%)
Sex	Female	4	7.3
	Male	51	92.7
Smoking History	Never Smoked	5	9.1
	Ex Smoker	48	87.3
	Active Smoker	2	3.6
Primary Tumor Location	Upper lobe of the right lung	19	34.5
	Lower lobe of the right lung	5	9.1
	Upper lobe of the left lung	15	27.3
	Lower lobe of the left lung	11	20.0
	Other	5	9.1
Primary Tumor Location	Peripheral	47	85.5
	Central	8	14.5
Endobronchial Lesion	Absent	52	94.5
	Present	3	5.5
Initial Stage	Stage 1–2	7	12.7
	Stage 3	16	29.1
	Stage 4	32	58.2
T Stage	T1	2	3.6
	T2	15	27.3
	T3	19	34.5
	T4	19	34.5
Nodal Status	N0	13	23.6
	N1	16	29.1
	N2	16	29.1
	N3	10	18.2
Mixed Histology	Absent	50	90.9
	Present	5	9.1
TTF-1	Negative	31	56.4
	Positive	23	41.8
	Unknown	1	1.8
Synaptophysin	Negative	10	18.2
	Positive	45	81.8
	Unknown	0	0.0
Chromogranin	Negative	24	43.6
	Positive	30	54.5
	Unknown	1	1.8

**Table 2:** Clinical and Demographic Findings-2.

Variables	Categories	*n*	%
M Stage	M0	23	41.8
	M1	32	58.2
Is there a history of surgery at the time of initial diagnosis?	No	36	65.5
	Yes	19	34.5
History of Postoperative Chemotherapy	No	36	65.5
	Yes	19	34.5
History of Postoperative Radiotherapy	No	51	92.7
	Yes	4	7.3
Is there CNS metastasis in the diagnosis?	No	46	83.6
	Yes	9	16.4
Is there liver metastasis in the diagnosis?	No	43	78.2
	Yes	12	21.8
Is there bone metastasis at diagnosis?	No	36	65.5
	Yes	19	34.5
Is there adrenal metastasis in the diagnosis?	No	46	83.6
	Yes	9	16.4
Is there lymph node metastasis at diagnosis?	No	20	36.4
	Yes	35	63.6
First Line Treatment Regimen Protocol	SCLC	40	83.3
	NSCLC	8	16.7
Second Line Treatment Regimen Protocol	SCLC	11	64.7
	NCSLC	6	35.3
First Line Treatment Regimen	Platin + Etoposide	40	72.7
	Other Treatments	8	14.5
	Treatment refusal or ex	7	12.7
Imaging Method Used in Follow-up	Unknown	6	10.9
	Conventional	1	1.8
	PET-CT	48	87.3
First Line Recist Best Response	CR	5	9.1
	PR	8	14.5
	SD	1	1.8
	PD	20	36.4
	Unknown	21	38.2
First-line treatment discontinuation reason	did not receive treatment	7	12.7
	Progression (including ex patient)	31	56.3
	Unknown	4	7.3
	Completed the Treatment	11	20.0
	Treatment Continues	2	3.6
Second Line Treatment Regimen	Did not receive treatment/unknown	38	69.1
	Platin + Etoposide	5	9.1
	Irinotecan	3	5.5
	Carboplatin + Paclitaxel	5	9.1
	Topotecan	2	3.6
	Docetaxel	1	1.8
	Cisplatin + Irinotecan	1	1.8
Second Line Recist Best Response	CR	1	1.8
	SD	1	1.8
	PD	8	14.5
	Unknown	45	81.8
Follow-up Situation	Unfollowed	22	40.0
	Followed	33	60.0
Current Situation	Alive	12	21.8
	Exitus	43	78.2

M Stage: Metastatic Stage; SCLC: Small Cell Lung Cancer; NSCLC: Non-Small Cell Lung Cancer; PET-CT: Positron Emission Tomography–Computed Tomography; CR: Complete Response; PR: Partial Response; SD: Stable Disease; PD: Progressive Disease.

**Table 3:** Survival Times of Patients According to Initial Stage.

Initial Stage	N	mOS (Months)	95% CI (Months)
Stage 1–2	7	NR	12-NR
Stage 3	16	9	5–16
Stage 4	32	7	2–9

NR: Not Reached.

**Table 4:** Survival times according to tumor location and other characteristics.

	Categories	N	mOS (Months)	95% CI (Months)	P (Log Rank)
Age	<65	30	14	10–20	<0.01
	≥65	25	5	3–7	
T Stage	T1	2	NR	-	0.045
	T2	15	11	2-NR	
	T3	19	9	5–14	
	T4	19	7	3–9	
N Stage	N0	13	13	4-NR	0.172
	N1	16	10	4–12	
	N2	16	9	5–14	
	N3	10	2	1–9	
TTF-1	Negative	31	8	4–10	0.092
	Positive	23	11	5–38	
Synaptophysin	Negative	10	7	4–14	0.639
	Positive	45	9	5–12	
Chromogranin	Negative	24	8	4–16	0.977
	Positive	30	9	5–11	
The Best Treatment Response (RECIST)	CR	5	NR	13-NR	0.0005
	PR	8	7	3-NR	
	SD	1	NR	-	
	PD	20	10	7–14	
Could not be evaluated (Recist)		21	4	1–8	

NR: Not Reached; TTF-1: Thyroid transcription factor-1; CR: Complete Response; PR: Partial Response; SD: Stable Disease; PD: Progressive Disease.

## Data Availability

The datasets generated and/or analysed during the current study are not publicly available due but are available from the corresponding author on reasonable request.
